# Pulmonary valve replacement after correction of Tetralogy of Fallot

**DOI:** 10.1186/1749-8090-8-S1-P176

**Published:** 2013-09-11

**Authors:** R Meza, C Obando, R Breciani, N Sandoval

**Affiliations:** 1 Congenital Heart Surgery, Fundacion Cardioinfantil, Bogota, Colombia

## Background

The Tetralogy of Fallot (TOF) is a frequent cardiopathy in our days, corresponding to 7% of the congenital cardiopathies.

However it is acknowledged that one of the main causes of reintervention in these patients is the presence right ventricular failure secondary to pulmonary valve insufficiency (IP) leading the patient to intolerance to exercise, ventricular tachycardia and sudden death. The aim of our investigation is describing our experience of those patients subjected to (TOF) correction who have required a pulmonary valve replacement.

## Methods and results

Between January 1996 and December 2011 252 individuals with Tetralogy of Fallot (TOF) were intervened. Of these, 10 patients (3, 9%) required replacement of pulmonary valve (5 men and 5 women).

The patients’ average age was 9, 3 ± 3, 9 years.

The average time interval between the (TOF) correction and the replacement of pulmonary valve was 7, 2 ± 3, 5 years.

The main indication for replacement of pulmonary valve was right cardiac failure 50% (5/10).

We used Contegra graft in 20% (2/10) patients, allograft in 40% (4/10) patients, bioprosthesis in 40% (4/10) patients.

As concomitant corrected procedures during the pulmonary valve replacement 30% (3/10) patients with residual interventricular communication and tricuspid valve repair in 40% (4/10) patients.

The average aortic clamping time was of 119.4 ± 62.5 minutes.

The average ICU stay was 3.5 ± 1, 5 days.

An early mortality was present 10% (1 patient) and no one presented late mortality.

In the postoperative echocardiographic controls was found a decrease of the right ventricle diameter 31,4 ± 7,9 vs 27,8 ± 4,8 and improvement of the ventricular function 69,9 ± 11,1% vs 72,2 ± 12%

## Conclusion

The pulmonary valve replacement after correction of (TOF) significantly improves the function of the right ventricle. It is a safe procedure conducted with a low mortality rate.

